# Dataset from healthy and defective spot welds in refill friction stir spot welding using acoustic emission

**DOI:** 10.1016/j.dib.2022.108750

**Published:** 2022-11-15

**Authors:** Fethi Dahmene, Slah Yaacoubi, Mahjoub El Mountassir, Gaëlle Porot, Mohamed Masmoudi, Pascal Nennig, Uceu Fuad Hasan Suhuddin, Jorge Fernandez dos Santos

**Affiliations:** a Equipe Monitoring et Intelligence Artificielle, Institut de Soudure, 4 boulevard Henri Becquerel, Yutz 57970, France; b Equipe CND avancés, Institut de Soudure, 4 boulevard Henri Becquerel, Yutz 57970, France; c Materials Mechanics, Solid State Joining Processes, Helmholtz-Zentrum Hereon, Institute of Materials Research, Max-Planck-St.1, Geesthacht 21502, Germany

**Keywords:** Refill friction stir spot welding, Process monitoring, Condition monitoring, Defect detection, Acoustic emission

## Abstract

The dataset presented in this paper deals with real-time measurements carried out during the welding of 78 spot welds including heathy and defective states. These measurements are composed of acoustic emission signals and welding parameters. Acoustic emission signals were captured by three different piezoelectric sensors, which are connected to a Vallen AMSY5 system through preamplifiers. Welding parameters where digitized using the M-SCOPE software. Both measurements can be used for the establishment of an automatic criterion able to detect defective spot welds in Refill Friction Stir Spot Welding.

**Specifications Table**

**Table:** 

Subject	Engineering
Specific subject area	Process monitoring of Refill Friction Stir Spot Welding (Refill FSSW)
Type of data	Table (TXT, CSV) Vallen files
How the data were acquired	Piezoelectric acoustic emission sensors and acoustic emission data acquisition. Welding parameters were acquired using M-SCOPE software.
Data format	Raw
Description of data collection	A database of 78 healthy and defective spot welds including various defects type, was produced. The spot welds are performed sequentially (i.e. one by one). Each spot weld was monitored using acoustic emission and welding parameters. Defective welds were carried out using intentionally slight parameters drift as follows: - Defective welds containing flash and lack of mixing: generated mainly during the calibration phase of the zero level (difference of burr level height between probe, shoulder, and base metal surfaces), - Defective welds containing incomplete refill and lack of mixing: using less plunge depth of 0.05 mm and 0.1 mm, - Defective welds containing contamination (presence of sealant inclusion in the joint): welding without squeeze-out phase. This additional phase consists of moving downward the tool and clamp the sheets several second to move the sealant out from the joining area. Data collection follows six main steps: experimental set-up preparation, welding, real-time monitoring, post-welding non-destructive characterization, destructive characterization (observation of cross-section), and finally welds classification. This latter was performed using non-destructive testing (NDT) and cross-section but it is out of the scope of this paper.
Data source location	Institut de Soudure 4 Boulevard Henri Becquerel 57,970 Yutz, FRANCE
Data accessibility	Repository name: Mendeley Data Data identification number: DOI: 10.17632/fmvc523fzc.1 Direct URL to data: https://data.mendeley.com/datasets/fmvc523fzc/1

**Value of the Data**

• The data are acoustic emission (AE) signals obtained during Refill FSSW of healthy and defective welds. It can be used as a benchmark to develop/validate real-time defective weld detection using AE technology or welding parameters.
• The data can be used to analyse the influence of spot weld state on AE signal or welding parameters.
• The data can be utilized to develop criteria for defect detection and more hopefully identification in Refill FSSW.
• Researchers can evaluate their Machine learning models on this data to 1/ detect defective welds, 2/ to identify defects, 3/ to classify the different defects in various classes.
• The process of Refill FSSW is somewhat emergent, and this kind of data is helpful, for designers, R&D researchers and data scientists, to work hand-on-hand, to gain on the technological readiness level of this process.

## 1 Data Description

The development of monitoring solutions in Refill FSSW is a key factor for the deployment and acceptance of this process in industry. Acoustic emission (AE) [1] monitoring is the most promising solution that can address this challenge thanks to its history and experience in welding [2,3], which are inspirational. Despite the important interest of researchers on the use of AE in similar process such as friction stir welding (FSW) [4,5], the technology readiness level still below industrial expectations. Indeed, the switch from healthy state to defective one is often based on the changing of welding parameters [6,7], which is not representative of industrial applications (fixed welding procedure). Hence, sharing and capitalizing a database of healthy and defective spot welds using a slight welding parameters drift will help the research community to faster the technology-readiness of process monitoring in Refill FSSW.

**Fig. 1:**
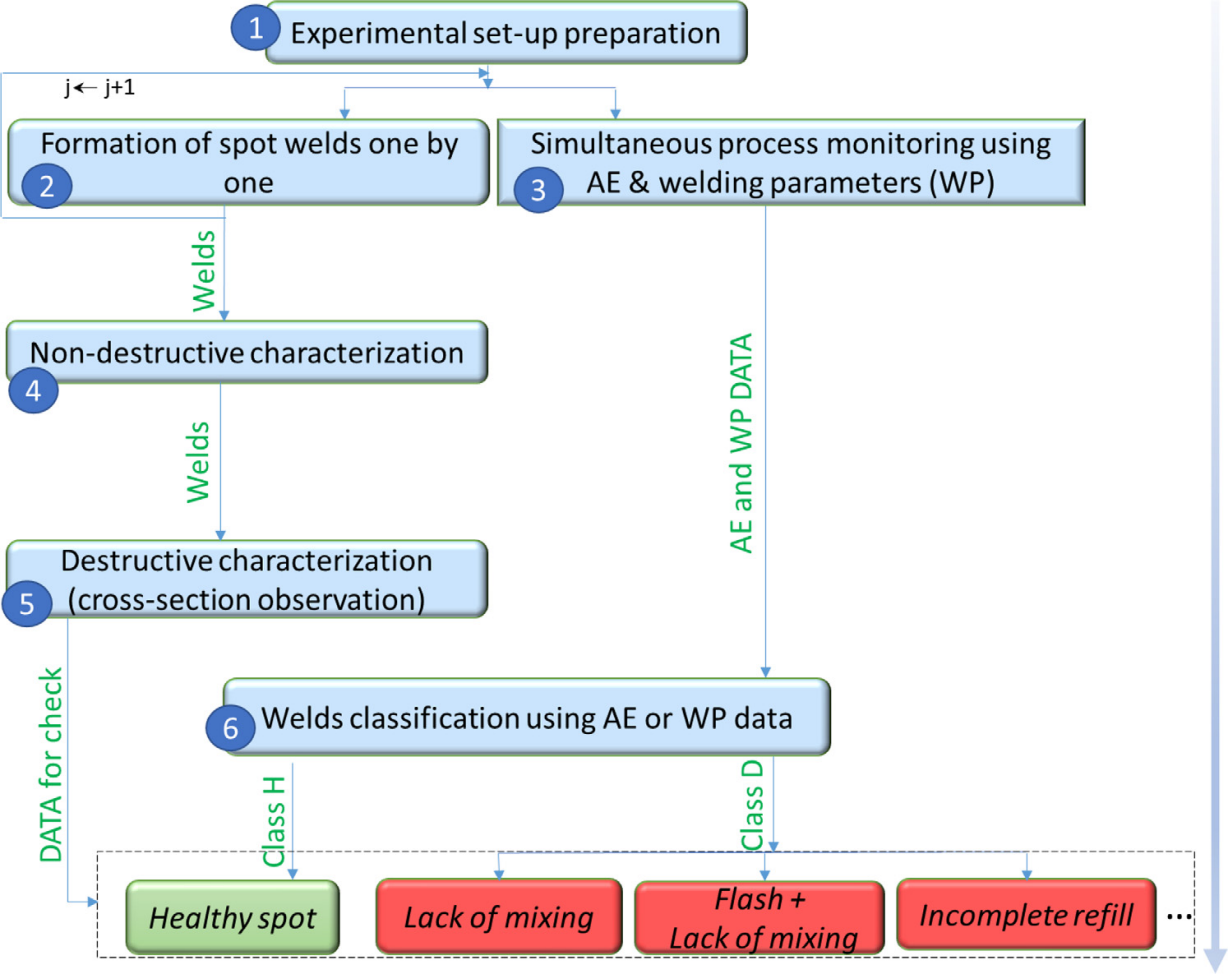
Flow chart of data generation. (1) Set-up preparation, (2–3) welding and simultaneous process monitoring, (4) Non-destructive characterization, (5) destructive characterization, (6) classification.

The collected database follows the flow chart of Fig. 1. As it can be noticed, six steps are implemented: Experimental set-up preparation, Welding, real -time monitoring with AE and welding parameters, non-destructive characterization, destructive characterization using cross-section observation, and finally the classification of spots welds.

The database of 78 healthy and defective spot welds including various defects type is reported in Table 1. The classification on healthy (H) or defective (D) is obtained using NDT and cross-section characterization. Characterization using macrography cross-section was carried out on some spot welds to consolidate NDT results.

**Table 1:** Database of the Refill FSSW, and their corresponding states using post-process NDT techniques and cross-section.

The data is classified in three folders. The first one named “Original AE data” contains files generated by the Vallen acquisition unit. The *.pridb file contains the AE-data acquired with the acquisition setup detailed in the next section. The *.tradb file contains the acquired waveforms. The *.vae file contains the analysis setup used during real-time monitoring. To open these files, researcher will need the vallen control panel release available in this link (www.vallen.de/downloads/). To facilitate the use of AE data, another folder named “Extracted AE database” is created, which contains extracted AE signal for the 78 spot welds. Data are TXT files. Some information is included in each file such as the sample rate, pre-trigger samples, the channel number (or sensor number) and the number of samples. The data starts from line 14 of the file, which is the amplitude in (mV). The time between two samples is calculated by inverting the sample rate (sampling frequency). The third folder is dedicated to the database of welding parameters monitoring. It is composed of 78 CSV files. Each file contains 8 welding parameters, which are defined as specified in Table 2.

**Table 2:** Welding parameters.

Relevant Process Data	Unit	Factor On Delivery (M-scope)	Parameter Name
Spindle Motor Speed	[1/min]	1	RPS100:1:RPS100/.QVW_650_AKT_SPEED
Spindle Active Current 100% Servo controller rate current =I_n_ AC 24 A (at 3 × 400 V grid)	[1000=100%]	1	RPS100:1:RPS100/.QVW_650_AKT_CURRENT
Sleeve Motor Speed	[1/min]	1	RPS100:1:RPS100/.QVW_670_AKT_SPEED
Sleeve Active Current 100% Servo controller rate current =I_n_ AC 4 A (at 3 × 400 V grid)	[1000=100%]	1	RPS100:1:RPS100/.QVW_670_AKT_CURRENT
Sleeve Stroke	[mm]	1000	RPS100:1:RPS100/.QVR_670_ISTPOS_mm
Sleeve Offset	[mm]	1000	RPS100:1HWHCAN02/.GR_670_Sleeve_F_Offset
Pin Motor Speed	[1/min]	1	RPS100:1:RPS100/.QVW_660_AKT_SPEED
Pin Active Current 100% Servo controller rate current =I_n_ AC 4 A (at 3 × 400 V grid)	[1000=100%]	1	RPS100:1:RPS100/.QVW_660_AKT_CURRENT
Pin Stroke	[mm]	1000	RPS100:1:RPS100/.QVR_660_ISTPOS_mm
Pin Offset	[mm]	1000	RPS100:1HWHCAN02/.GR_660_Pin_F_Offset
Clamp Offset	[mm]	1000	RPS100:1HWHCAN02/.GR_630_Clamp_F_Offset

## 2 Experimental Design, Materials and Methods

In this work, 0.6 mm-thick AA2024-T3 alloy specimens were used in Refill FSSW. Sheets dimensions are 120 mm × 30 mm and used as a band specimen or combined to form a lap shear specimen. This latter contains two sheets with 30 mm overlap and spot welded in the centre. All spot welds were carried out with a commercially RPS100® machine produced by Harms & Wende®. The tool is composed of a probe, a shoulder, and a clamping, with 4, 6, and 12 mm diameters, respectively. Welding used parameters are 1250 rpm for rotational speed (RS), 0.8 mm for plunge depth (PD), 0.7 mm/s for shoulder retracting/plunging rate or feeding rate (FR), and 10 kN for welding force.

**Fig. 2:**
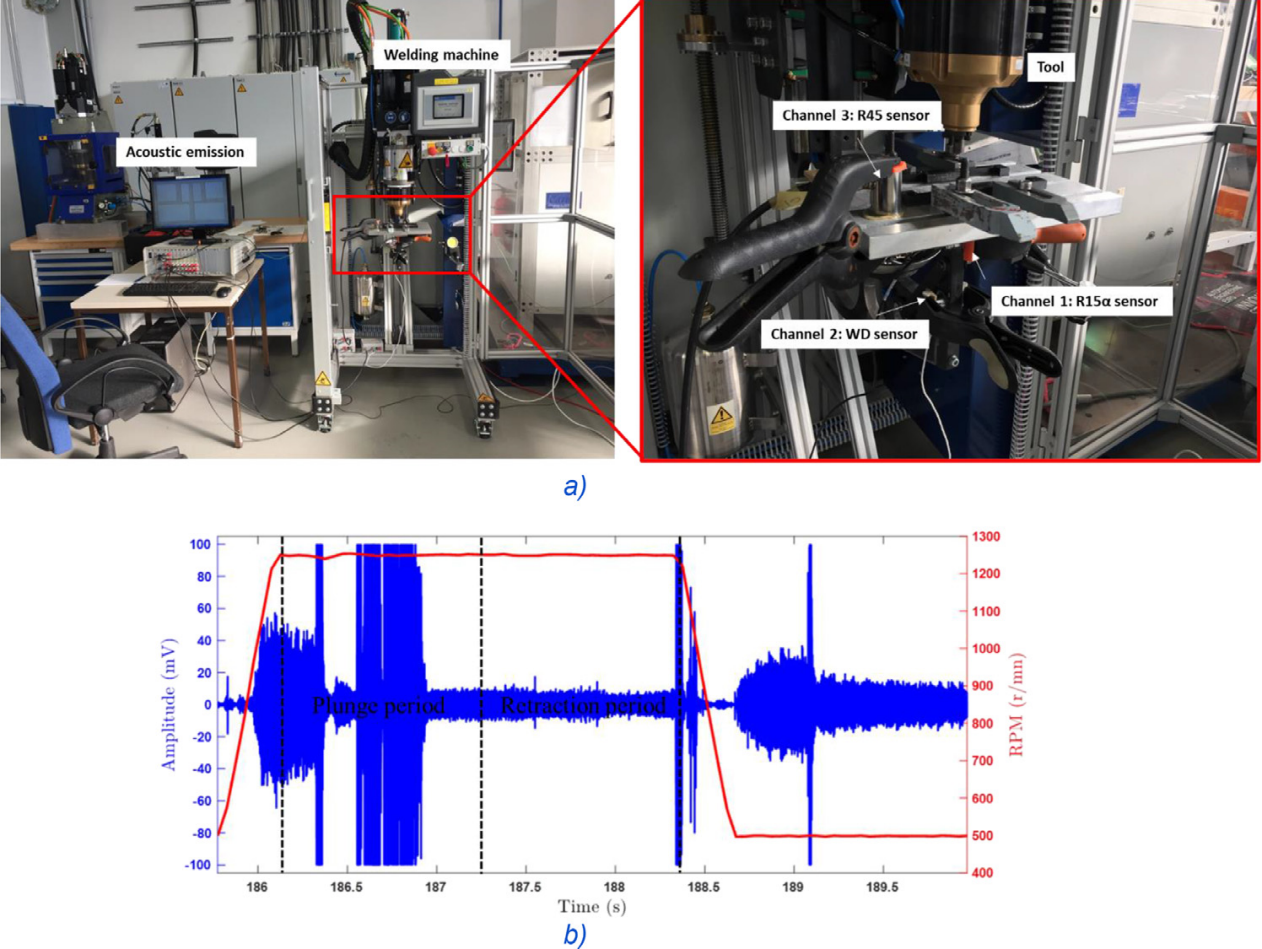
(a) Experimental setup. (b) Synchronization of AE signal with the rotation speed.

An advanced AE monitoring system, AMSY5, and three sensors located in different locations, as it can be seen in Fig. 2, were used. They are noted S_i_ / *i* = 1–3. Their respective commercial names are R15α, WD, and R45. Whole the sensors were acoustically coupled using a vacuum grease and fixed to the support using plastic clamp. The acquired AE signals were amplified by a 34 dB fixed gain using an AEP3N pre-amplifier. A relatively large pass-band hardware filter (i.e., 20–500 kHz) was used to detect a wide band of frequencies. The AE was recorded in the continuous mode at a sampling rate of 0.5 MSP/s (i.e., 0.5 mega samples per second).

The synchronization between an AE signal and the welding phases of each weld was performed using a custom developed trigger. It is based on a relay which is connected directly to the machine controller and more specifically to the rotation speed output. The trigger adapts (decreases) the peak controller voltage from 24 V to 10 V and delivers it to the AE system. The implementation of the conversion factor in the AE system (0.33 rpm/mv) allows to record the rotation speed with the AE signal and offers the possibility of triggering the region of interest. In our case, the AE signal was recorded once the rotation speed exceeds 500 rpm (Fig. 2). This enables to capture the AE signal from the first contact between the tool and the specimen.

Welding parameters have been monitored using the software M-SCOPE. It ensures data extraction and recording of the following parameters: pin stroke, sleeve stroke, spindle active current, pin active current, spindle motor speed, pin motor speed, sleeve motor speed, sleeve active current, and clamp offset. A csv file is then generated for each spot weld. Due to the manual character of welding parameter extraction, a time shift between spot welds is induced. This can be fixed by the application of data correction.

**Fig. 3:**
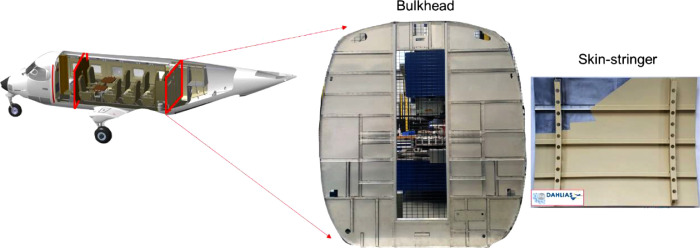
Manufactured demonstrator that was monitored using the developed method.

It is worth noting that the method developed here for the monitoring of the Refill FSSW process was reused later, in the same R&D project, as it stands for the manufacturing of the demonstrator shown in Fig. 3. All the desired data were collected without any problem; for example, the vibration of the machine did not have any impact on the quality of the data thanks to 1/ the good choice of the frequency and 2/ the placements of the sensors, the used coupling, and the manner of their attachment. In conclusion, the proposed method is reliable and basing on this, it can be applied *in-situ* as it stands, independently of the material type to be welded and the dimensions of the structure to be assembled.

## Ethics Statements

N/A.

## CRediT authorship contribution statement

**Fethi Dahmene:** Writing – original draft, Investigation, Data curation. **Slah Yaacoubi:** Conceptualization, Methodology, Supervision, Writing – review & editing. **Mahjoub El Mountassir:** Visualization, Investigation, Formal analysis. **Gaëlle Porot:** Investigation, Funding acquisition, Project administration. **Mohamed Masmoudi:** Investigation. **Pascal Nennig:** Investigation, Funding acquisition, Project administration. **Uceu Fuad Hasan Suhuddin:** Methodology, Resources, Conceptualization. **Jorge Fernandez dos Santos:** Resources, Funding acquisition, Project administration.

## Declaration of Competing Interest

The authors declare that they have no known competing financial interests or personal relationships that could have appeared to influence the work reported in this paper.

## Data Availability

AE monitoring of Refill FSSW (Original data) (Mendeley Data).
